# A comparison of asymmetric dimethylarginine, arginine, galectin-3, and echocardiographic data with bipolar disorder and schizophrenia

**DOI:** 10.1590/1806-9282.20241948

**Published:** 2025-07-07

**Authors:** Gökçe Kavak Sinanoğlu, Memduha Aydın, Kürşat Altınbaş, Sedat Abuşoğlu, Kenan Erdem

**Affiliations:** 1 ALKU Alanya Education and Research Hospital, Department of Psychiatry – Antalya, Türkiye.; 2 Selçuk University, Department of Psychiatry – Konya, Türkiye.; 3 Private Clinic, Department of Psychiatry – İstanbul, Türkiye.; 4 Selçuk University, Department of Biochemistry – Konya, Türkiye.; 5 Selçuk University, Department of Cardiology – Konya, Türkiye.

**Keywords:** Schizophrenia, Bipolar disorder, Arginine, ADMA, Galectin-3, Echocardiography

## Abstract

**OBJECTIVE:**

Cardiovascular mortality in schizophrenia and bipolar disorder patients is more than double that of the general population. There appears to be a dual effect between cardiovascular diseases and schizophrenia and bipolar disorder. Inflammation and increased oxidative stress cause cardiac remodeling and increase the severity of psychiatric illness.

**METHODS:**

Echocardiographic imaging was performed simultaneously with serum samples obtained for asymmetric dimethylarginine, symmetric dimethylarginine, monomethyl-L-arginine, arginine and its metabolites, and galectin 3 levels in 80 patients with bipolar disorder who were euthymic for at least 8 weeks and 69 patients with schizophrenia undergoing treatment.

**RESULTS:**

Monomethyl-L-arginine and arginine levels were significantly higher in bipolar disorder patients compared to schizophrenia patients. Galectin 3 level was significantly higher in bipolar disorder patients with psychotic symptoms compared to schizophrenia patients. There was no significant difference between the groups in terms of other parameters. Statistically significant differences were found between bipolar disorder and schizophrenia patients in terms of diastolic diameter (mm), systolic diameter (mm), interventricular septum (mm), left atrium (mm), and posterior wall (mm) values.

**CONCLUSION:**

Arginine and its metabolites, asymmetric dimethylarginine, symmetric dimethylarginine, and galectin 3, may be important biomarkers of cardiovascular diseases risk. In this study, changes in biochemical parameters and cardiac structure could not be explained by disease severity.

## INTRODUCTION

Life expectancy in patients with schizophrenia and bipolar disorder is approximately 15–25 years shorter than in the general population^1^. Smoking, sedentary lifestyle, inadequate self-care, negative symptoms, cognitive impairment, and side effects of antipsychotics cause high rates of CD in patients with SCZ and BD^2^. Additionally, sleep disorders, obesity, diabetes, and a weakened immune system may contribute to the exacerbation of the existing mental illness^3^. Due to this dual effect between psychiatric illnesses and CD^4^, many studies have aimed to reduce cardiovascular mortality with biomarkers and imaging methods.

Asymmetric dimethylarginine (ADMA) is an endogenous inhibitor of nitric oxide (NO) production^5^. Monomethyl-L-arginine (L-NMMA), asymmetric dimethylarginine (ADMA), and symmetric dimethylarginine (SDMA) are formed by methylation of L-arginine. ADMA decreases NO formation in a concentration-dependent manner. Reduced NO production leads to a shift in the homeostatic vascular balance between vasodilation and vasoconstriction. It may contribute to the proinflammatory state of the vessel wall, thus triggering atherosclerosis^6^. Methylated analogs of the amino acid L-arginine have gained major importance as prognostic biomarkers for CD and mortality^7^. Additionally, NO stimulates the synaptic release of neuromodulators and monoamines in the central nervous system. It has been reported that ADMA plays a role in the regulation of the release of molecules such as acetylcholine, dopamine, GABA, and glutamate through neural membrane depolarization^8^ and reduces cerebral blood flow through vasocontraction as a result of NOS inhibition, causing psychiatric diseases^9^.

Gal-3, a potent inflammatory protein, may contribute to the initiation and amplification of both acute and chronic inflammation^10^. Inflammation has been shown to play an important role in the development and severity of SCZ and BD^11^. The findings suggest that Gal-3 may possess systemic inflammatory properties, potentially mediated through its interactions with proinflammatory markers that contribute to immunometabolic processes in SCZ. It has been reported that somatic comorbidities commonly associated with SCZ, such as obesity, hyperlipidemia, and type 2 diabetes, could be monitored by measuring Gal-3^12^. However, there are few studies in the literature investigating how Gal-3 levels change in psychiatric disorders. The association of high Galectin-3 with heart failure and CD has led to the use of Gal-3 levels as a biomarker for CD risk^13^.

The aim of this study was to evaluate and compare the risk of cardiovascular disease in patients with schizophrenia and bipolar disorder using biochemical parameters and echocardiography imaging methods to assess the risk of cardiovascular disease.

## METHODS

The study included 80 BD, who were diagnosed according to DSM-5 diagnostic criteria, whose disease diagnosis was confirmed by at least two specialists, and who were in remission for the last 8 weeks, and 69 SCZ patients who were not in a psychotic period. The patients included in the study were selected from among the patients who applied to the Psychiatry Department of Selçuk University Faculty of Medicine Hospital in 2021–2022.

Patients with chronic hypertension and blood pressure that cannot be controlled with treatment, and those with an ejection fraction below 50%, were excluded from the study. A tube of blood was collected from the volunteers included in the study to measure arginine and arginine metabolites, L-NMMA, SDMA, ADMA, and galectin-3, and echocardiography was performed by a cardiologist simultaneously.

### Clinical assessment scales

#### Echocardiographic data

As recommended by the American Society of Echocardiography for transthoracic studies, a transthoracic echocardiogram was performed by a cardiologist experienced in ultrasonography using a special ultrasound machine (Vivid E9, GE Vingmed, Horten, Norway) with an M5S cardiac sector probe (1.5–4.5 MHz).

#### Biochemical measurements

ADMA, SDMA, L-NMMA, arginine, citrulline, ornithine, homoarginine, and methylarginine levels were measured in serum samples taken from patients in the Biochemistry Metabolism Laboratory using liquid chromatography-tandem mass spectrometry (LC-MS/MS ABSCIEX API 3200) and electrospray ionization (ESI method). Galectin-3 and nitrite/nitrate tests were performed with a commercial ELISA kit.

### Statistical analysis

Data were analyzed using SPSS 21.0^®^. Data are presented as median (minimum–maximum). Normality was confirmed by the Kolmogorov-Smirnov test. The Kruskal-Wallis test and one-way ANOVA were applied in comparisons involving more than two groups. Mann-Whitney U test was applied in post-hoc analyses with Bonferroni correction to determine significant measurements. Tukey and Tamhane corrected post-hoc analyses were performed according to the homogeneity test. The Mann-Whitney U test was used in comparisons between groups. A Pearson correlation test was used to determine whether there was a relationship between biochemical data and echocardiography data. p<0.05 was considered statistically significant.

## RESULTS

### Demographic and clinical characteristics of participants

Notably, 80 BD and 69 SCZ patients completed the study. There were 38 male and 42 female patients with BD, and there were 39 male and 30 female patients with SCZ. Thirty-eight (47.5%) of the patients with bipolar diagnosis had experienced an episode with psychotic symptoms in the past. The mean age of BD was 36.16±12.73 years and SCZ was 36.79±12.09 years.

There was a statistically significant difference between BD and SCZ patients in L-NMMA and arginine parameters. This difference was between BD patients without psychotic features and SCZ patients. L-NMMA and arginine levels were significantly higher in BD patients compared to SCZ. Gal-3 levels were significantly higher in BD with psychotic symptoms compared to SCZ. No significant difference was found between the groups in terms of other parameters. The comparison of serum arginine, its metabolites, L-NMMA, SDMA, and ADMA values between the groups is given in Table 1.

**Table 1:** Comparisons of serum arginine, its metabolites, monomethyl-L-arginine, symmetric dimethylarginine, and asymmetric dimethylarginine measurement between groups.

Parameters	Non-psychotic BD	Psychotic BD	Schizophrenia	p-value	Post-hoc		
					Non-psychotic BD-Psychotic BD (p-value)	Non-psychotic BD-SCZ (p-value)	Psychotic BD-SCZ (p-value)
ADMA^a^	0.27 (0.11–0.62)	0.27 (0.130–0.508)	0.23 (0.068–0.441)	0.380*	0.847	0.317	0.202
SDMA^a^	0.45 (0.16–0.89)	0.46 (0.16–0.89)	0.39 (0.219–0.910)	0.129*	0.900	0.050	0.186
L-NMMA^b^	0.028 (0.09–0.058)	0.027 (0.012–0.062)	0.015 (0.004–0.027)	**<0.001** **	0.990	**<0.001**	**<0.001**
Ornıthıne^a^	29.46 (6.44–77.20)	22.96 (5.35–62.20)	26.50 (8.28–73.6)	0.072*	0.078	0.964	0.026
Styrulıne^a^	83.15 (23.7–171)	88.15 (19.8–266)	95.11 (36–199)	0.148*	0.682	0.086	0.143
Argınıne^b^	133.57 (42.8–313.7)	125.31 (32.1–193.8)	86.91 (15.3–200)	**<0.001** **	0.698	**<0.001**	**<0.001**
Homoarginine^a^	1.94 (0.72–4.04)	1.79 (0.52–4.81)	2.08 (0.178–6.29)	0.480*	0.405	0.720	0.239
Galectin 3^a^	903.07 (107.89–2,932.35)	856.22 (173.81–2,189.67)	716.43 (87.59–3,540.412)	**0.021** *	0.600	0.055	**0.010**

Note:

Values are presented as median (minimum–maximum).

* Kruskall-Wallis;

** One-way ANOVA.

b Parametric distribution;

a Non-parametric distribution.

Statistically significant values are indicated in bold. ADMA: asimetrik dimetilarginin; SDMA: simetrik dimetilarginin; L-NMMA: monometil-L-arginin; BD: bipolar disorder; SCZ: schizophrenia.

As a result of echocardiography performed on the volunteers who participated in the study, statistically significant differences were found between BD and SCZ patients in terms of diastolic diameter (mm), systolic diameter (mm), interventricular septum (mm), posterior wall (mm), and left atrium (mm) values. No significant difference was found between the groups in terms of other parameters. A comparison of echocardiography parameters between the groups is given in Table 2.

**Table 2:** Comparisons of echocardiography parameters between groups.

Parameters	Non-psychotic BD	Psychotic BD	Schizophrenia	p-value	Post-hoc		
					Non-psychotic BD-psychotic BD	Non-psychotic BD-SCZ	Psychotic BD-SCZ
Diastolic diameter^a^ (mm)	48.73 (45–55)	48.44 (42–59)	46.33 (40–53)	**<0.001** *	0.594	**<0.001**	**0.005**
Systolic diameter^a^ (mm)	30.59 (22–37)	30.55 (24–49)	28.40 (22–35)	**<0.001** *	0.499	**<0.001**	**0.007**
Interventricular septum^a^ (mm)	10.10 (8–11)	10.07 (8–14)	9.6 (7–12)	**0.001** *	0.851	**<0.001**	**0.007**
Posterior wall^a^ (mm)	9.83 (8–10)	9.73 (8–12)	9.50 (8–10)	**0.003** *	0.424	**0.001**	**0.041**
Left atrium^a^ (mm)	3.68 (2.60–4.40)	3.66 (2.80–4.80)	3.36 (2.60–4.60)	**<0.001** *	0.596	**<0.001**	**0.005**
Mitral E peak^b^ (cm/s)	81.95 (50–135)	80.32 (45–135)	75.98 (40–130)	0.219**	0.700	0.096	0.251
Mitral A peak^a^ (cm/s)	70.24 (45–105)	70.05 (45–125)	70.60 (40–120)	0.957*	0.809	0.959	0.784
Mitral E/A ratio^b^	1.21 (0.07–2.25)	1.19 (0.67–2.17)	1.11 (0.50–1.80)	0.264**	0.794	0.129	0.237
Ejection fraction^a^ (%)	59.33 (58–60)	59.55 (55–60)	59.62 (55–60)	0.076*	0.158	0.028	0.612

Note:

Values are presented as median (minimum–maximum).

* Kruskall-Wallis;

** One-way ANOVA.

b Parametric distribution;

a Non-parametric distribution.

Statistically significant values are indicated in bold. E/A ratio: ratio of the early (E) to late (A); BD: bipolar disorder; SCZ: schizophrenia.

The correlation analysis between the echocardiography parameters and biochemical parameters of the volunteers who participated in the study was conducted. There is a negative correlation between ADMA (r=-0.188; p<0.01) and EF; a negative correlation between L-NMMA (r=-0.201; p<0.01) and EF; a positive correlation between ADMA (r=0.186; p<0.01), L-NMMA (r=0.311; p<0.01), SDMA (r=0.222; p<0.01), and arginine (r=0.163; p<0.01) and left atrial diameter; L-NMMA (r=0.168; p<0.01) and SDMA (r=0.172; p<0.01) and interventricular septum (IVS); SDMA (r=0.170; p<0.01) and posterior wall (PW); and L-NMMA (r=0.164; p<0.01) and mitral E peak. The correlation analysis between the echocardiography parameters and the biochemical parameters of the participants is presented in Table 3.

**Table 3:** Correlations between biochemical parameters and echocardiography data.

Parameters	ADMA	LNMMA	SDMA	Ornıthıne	Styrulıne	Arginine	HArg	Gal3
Diastolic diameter	0.029	0.152	0.083	0.073	-0.129	0.109	-0.044	-0.044
Systolic diameter	0.072	0.146	0.088	0.088	-0.108	0.088	-0.040	-0.041
Interventricular septum	0.052	0.168*	0.172*	0.144	0.121	0.080	0.017	-0.136
Posterior wall	0.068	0.118	0.170*	0.121	0.151	0.072	-0.013	-0.157
Ejection fraction	-0.188*	-0.201*	-0.128	-0.027	-0.013	-0.080	-0.062	-0.033
Left atrium diameter	0.186*	0.311**	0.222**	0.124	-0.114	0.163*	-0.018	-0.029
Mitral E peak	-0.011	0.164*	0.004	-0.082	-0.120	0.059	-0.061	0.122
Mitral A peak	-0.007	-0.035	0.054	-0.022	0.085	-0.017	-0.022	-0.069
Mitral E/A ratio	0.013	0.184*	-0.024	-0.027	-0.159	0.081	-0.056	0.134

Pearson correlation coefficient:

* 0.05,

** 0.01.

ADMA: asymmetric dimethylarginine; L-NMMA: monomethyl-L-arginine; SDMA: symmetric dimethylarginine; HArg: homoarginine; Gal-3: galectin 3; E/A ratio: ratio of the early (E) to late (A).

## DISCUSSION

### Serum arginine, its metabolites, monomethyl-L-arginine, symmetric dimethylarginine, asymmetric dimethylarginine, and galectin-3 measurement

ADMA decreases in cerebral blood flow may cause the emergence of psychiatric illnesses^9^. Many studies report that ADMA and its metabolites are high in psychiatric diseases^14^. It was reported that ADMA levels decreased after treatment^15^ and ADMA levels increased with increasing disease burden^16^ and prolonged disease duration^9^. In a study comparing patients with SCZ experiencing an acute psychotic exacerbation and patients with BD in a manic episode to healthy controls, ADMA, SDMA, and L-arginine levels were found to be significantly higher in both SCZ and BD patients compared to the control group. However, no significant difference was observed between the patient groups. This finding has been attributed to potential impairments in antioxidant mechanisms in patients with SCZ and BD^14^. In our study, no significant differences were found between the groups in terms of ADMA levels. However, arginine and L-NMMA were statistically higher in patients with BD. Contrary to the existing literature, our findings did not explain disease severity in relation to arginine, its metabolites, and ADMA levels. This may be due to the fact that both disorders can lead to chronic inflammation through similar pathophysiological mechanisms^17^ share similar pharmacological treatments and that these medications exert comparable effects, particularly on the immune-inflammatory system^18^. Additionally, the small sample size, the heterogeneous nature of the disorders, and the influence of confounding factors such as body mass index and systemic diseases on ADMA may have contributed to this finding.

In our study, Gal-3 levels were found to be lower in patients with SCZ in remission compared to those with BD in remission. Consistent with our findings, a previous study reported lower serum Gal-3 in patients with SCZ compared to healthy controls^19^. Reduced serum Gal-3 concentrations have been suggested to indicate inflammation, pro-apoptotic activation, and impaired neurodegeneration in SCZ. Particularly in patients with predominant negative symptoms, decreased physical activity may contribute to lower Gal-3^19^. However, Kajitani et al. reported elevated serum Gal-3 in chronic SCZ^20^. In remitted schizophrenia patients, Gal-3 levels were found to be higher than in patients with first-episode psychosis and those experiencing a recurrence of psychotic symptoms. This study suggested that Gal-3 acts as a proinflammatory lectin in SCZ and that its elevation in chronic SCZ may contribute to myocardial fibrosis and metabolic alterations. Furthermore, Gal-3 may serve as a mediator in the underlying mechanisms of cardiovascular and metabolic changes in SCZ^21^.

### Echocardiography parameters

In our study, the diastolic diameter (mm), systolic diameter (mm), interventricular septum (mm), and left atrium (mm) measurements were significantly higher in patients with BD compared to those with SCZ. A previous study reported that patients with SCZ exhibited significantly smaller left ventricular and right ventricular end-diastolic volumes. Additionally, increased left ventricular concentricity and septal thickness in SCZ were suggested to indicate cardiac remodeling, potentially driven by systemic inflammation and pro-fibrotic factors^22^. In patients with BD, interventricular septal thickness and mean left ventricular end-diastolic diameter were found to be higher compared to healthy controls^23^. In a study, changes in heart structure were observed in SCZ and BD patients compared to healthy controls, and this was not found to be related to disease severity, as in our study^24^. Cardiac fibroinflammatory changes induced by elevated inflammatory cytokines and collagen accumulation may contribute to structural cardiac remodeling^22^.

## CONCLUSION

This study did not find a relationship between disease severity and structural cardiac changes based on the assessment of arginine and its metabolites, ADMA, Galectin-3 levels, and echocardiographic evaluation.

One of the strengths of this study is its comprehensive examination by simultaneously including multiple biochemical parameters and echocardiography to assess cardiovascular disease risk. Studies in this field in the literature often compared patient groups with healthy controls or evaluated attack periods. This study is valuable because it is the first to compare SCZ and BD patients in remission.

The limitations of the study include the small sample size, the absence of a healthy control group, the lack of sociodemographic data, and the lack of evaluation of factors that may affect the risk of cardiovascular disease, such as body mass index and smoking.

## Data Availability

The datasets generated and/or analyzed during the current study are available from the corresponding author upon reasonable request.
